# Identification and characterization of six novel tetraspanins from *Schistosoma japonicum*

**DOI:** 10.1186/1756-3305-4-190

**Published:** 2011-09-29

**Authors:** Yanyan Jiang, Xindong Xu, Xiaoxing Qing, Weiqing Pan

**Affiliations:** 1 Institute for Infectious Diseases & Vaccine Development, Tongji University School of Medicine, 1239 Siping Road, Shanghai 200092, PR China; 2 Department of Pathogen Biology, Second Military Medical University, 800 Xiang Yin Road, Shanghai 200433, PR China

## Abstract

**Background:**

Tetraspanins (TSPs), also known as members of the trans-membrane 4 super-family (TM4SF), comprise an assemblage of surface antigens reported in eukaryotic organisms. In the work presented here, six novel TSP proteins from the human blood fluke *Schistosoma japonicum *(*S. japonicum*) were produced and analyzed through a combination of bioinformatics and experimental approaches.

**Results:**

Six novel TSP proteins of *Schistosoma japonicum *(designated as Sj-TSP-#1~6) contained four trans-membrane regions and one large extracellular loop (LEL) with a conserved CCG motif. Size of the proteins varied from 227 to 291 amino acid residues. All the six proteins were produced in *E.coli *and immune sera to each protein were prepared. Analysis of transcription profiles of the proteins by RT-PCR showed that Sj-TSP-#4 was transcribed only in the egg stage while transcription of the Sj-TSP-#2 was detected in female worms but not in males. The similar results were obtained by Western blot. Immunolocalization of the TSP proteins by immunofluorescence assay showed that the Sj-TSP-#2, Sj-TSP-#5 and Sj-TSP-#6 were located in the tegument of worms.

**Conclusions:**

This study provided six novel TSP members of *S. japonicum *including their sequences and recombinant proteins. Availability of the novel proteins and information on their expression profile and location provided a basis for further investigation of the TSP proteins for their biological functions and as vaccine candidates.

## Background

Tetraspanins (TSPs) are members of the trans-membrane-4-superfamily (TM4SF) and are widely expressed in eukaryotic organisms. The first reported member of the TM4SF, CD81, was recognized when murine mAb-5A6 reacted proteins were studied in a human cell line [1]. After the genomic sequencing of several model organisms, numerous TSP members were found and proved to be conserved through distantly related species by expressed sequence tag analysis [2]. The TSP family includes 33 mammalian members [3,4], at least 37 members found in the *Drosophila melanogaster *genome [5] and 20 members in the *Caenorhabditis elegans *genome [6,7]; the family also includes fungus [8] and sponge TSPs [9].

Members of the family are characterized by the following four trans-membrane regions: short cytoplasmic tails at the N- and C-terminus, the small extracellular loop (SEL), the large extracellular loop (LEL) with a conserved Cys-Cys-Gly (CCG) motif and other juxtamembrane cysteine residues of palmitoylation sites [10]. The physiological roles of TSP are largely unknown. Recently, several lines of evidence indicated that TSP is involved in cell adhesion, migration, tumor metastasis and neurite outgrowth [11,12]. TSP also has been reported to interact with many proteins that are critically important to immune function, such as forming immune-complex to regulate B cells proliferation, binding B cell or T cell epitope to lead to polarization towards a Th1 response, re-arranging distribution of MHC II molecules to modulate peptide loading and etc [7,12-14].

At present, several members of the TSP family have been identified in *Schistosoma mansoni *(*S. mansoni*) and *Schistosoma japonicum *(*S. japonicum*) such as Sj25 [15], SjTE736 [15], Sm23 [16]/Sj23 [17], SmTSP1 [18] and SmTSP2 [18]/SjTSP2 [19,20]. According to the numbers of TSP from the above species, other members of this family should exist in *S. japonicum*. To further investigate the sequences of TSP members and their potential functions, it is necessary to identify and analyze other new TSP members. Therefore, in the present study, we screened for new members of TSP using bioinformatics and experimental approaches in *S. japonicum*. In addition, the putative TSP proteins were produced and analyzed for their expression profiles at transcriptional and translational level among various developmental stages and genders of the parasite.

## Results

### Identification of novel TSP members in *S. japonicum*

**Figure 1:**
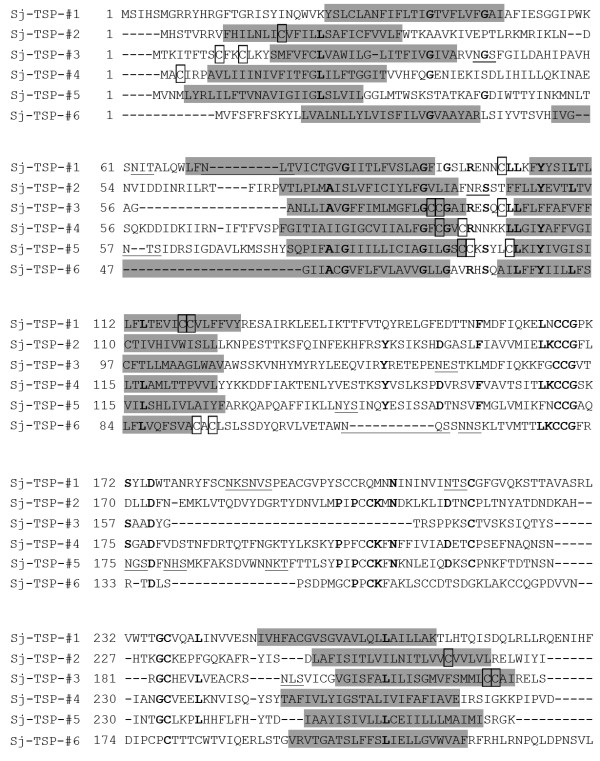
**Amino acid sequence alignment of six *S. japonicum *TSP proteins**. The four putative transmembrane domains are indicated by a grey shadow. The residues in bold type are fully conserved among the six TSP sequences. The juxtamembrane cysteine residues of palmitoylation sites are boxed. Potential sites of N-glycosylation are underlined.

Structure characteristic of 'tetraspanin' proteins contain four trans-membrane regions, forming two loops including a SEL and a LEL which contains a conserved CCG motif located at the C-terminus [21]. According to the protein structures, we used TMHMM2.0 software to screen for 523 sequences from integrated information of transcriptome and proteome of *S. japonicum *[22], we identified six novel TSP proteins which were designated as Sj-TSP-#1~6 (Table 1). Alignments of the deduced amino acid sequences of the six novel TSP proteins were shown in Figure 1. All the proteins contain the conserved 'CCG motif' indicated in bold and four putative hydrophobic trans-membrane regions. Moreover, the TSP proteins also contain several different predicted palmitoylation sites and N-glycosylation sites (Figure 1).

**Table 1:** Characteristics of the six TSP proteins in *S. japonicum.*

Serial Number	Cluster	Accession Number	Annotation	Size of predictive Protein (aa)	N-terminal Position	SEL Position	Inside Position	LEL Position	C-terminal Position
**Sj-TSP-#1**	SJCHGC07033	AY815196	>gb|AAP05961.1| similar to NM_079585 tetraspanin 86D in Drosophila melanogaster [Schistosoma japonicum]	291	1-26	50-68	92-103	127-248	272-291
**Sj-TSP-#2**	SJCHGC00527	AY813090	SJCHGC00527 protein[Schistosoma japonicum], similar to AAP05974-hypothetical protein(Schistosoma japonicum)	275	1-8	32-69	93-98	122-245	269-275
**Sj-TSP-#3**	SJCHGC02844	AY813131	SJCHGC02844 protein[Schistosoma japonicum], similar to AAH71132-Unknown (protein for MGC:82181)(Xenopus laevis)	227	1-15	39-57	81-86	110-200	224-227
**Sj-TSP-#4**	SJCHGC02880	AAP05954.1	>gb|AAP05954.1| hypothetical protein, putative CD9/CD37/CD63 antigens [Schistosoma japonicum]	282	1-6	30-74	98-103	127-246	270-282
**Sj-TSP-#5**	SJCHGC06407	AY815442	SJCHGC06407 protein[Schistosoma japonicum], similar to AAP05974-hypothetical protein(Schistosoma japonicum)	274	1-4	28-78	99-104	128-247	271-274
**Sj-TSP-#6**	SJCHGC02280	AY814594	SJCHGC02280 protein[Schistosoma japonicum], similar to CG6323-PA(Drosophila melanogaster)	233	1-11	35-43	67-72	93-194	218-233

**Figure 2:**
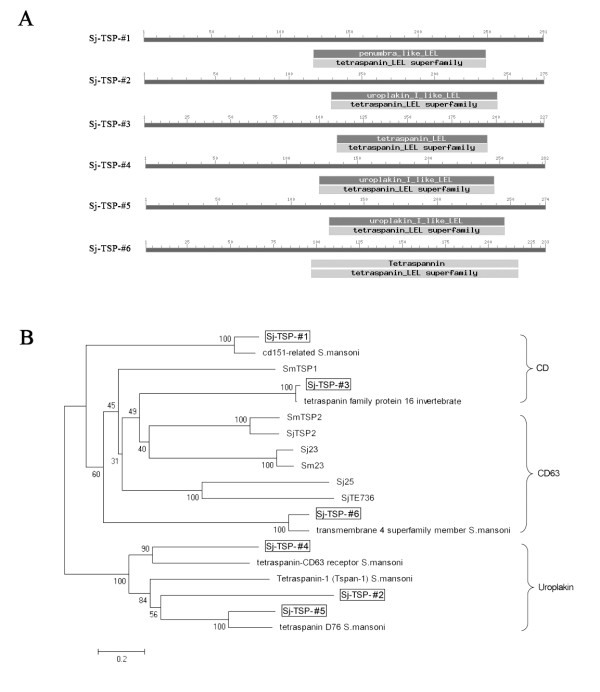
**Characteristic motifs and phylogenetic analysis of the six TSP proteins**. A: The domains of the TSP proteins were predicted by using CD-Search software. Heavy grey is a higher specific hit while bright grey is that they all contain a tetraspanin-like superfamily domain. B: Homologue phylogenetic tree of sequences from *S. mansoni *and Sj-TSP-#1~6 (in marked frame) created with the use of MEGA4. These sequences could be subdivided into three major monophyletic subfamilies (the CD family, the CD63 family and the Uroplakin family). Accession number of these sequences: cd151-related [Genbank: XP_002582093.1], Tetraspanin-1 (Tspan-1) [Genbank: XP_002577444.1], tetraspanin family protein 16 invertebrate [Genbank: XP_002573925.1], tetraspanin-CD63 receptor [Genbank: XP_002574749.1], tetraspanin D76 [Genbank: XP_002575497.1], transmembrane 4 superfamily member [Genbank: XP_002579012.1], SjTE736 [Genbank: AAC69992.1], SjTSP2 [Genbank: AEG74364.1], Sj25 [Genbank: AAB88626.1], Sj23 [Genbank: AAA29920.1], Sm23 [Genbank: P19331.1], SmTSP1 [Genbank: AAN17278], SmTSP2 [Genbank: AAN17276.1].

We also predicted putative domains in the six TSP proteins by searching a conserved domain database (CDD). As shown in Figure 2A, Sj-TSP-#1 shared many specific matches with penumbra [23], a member of TM4SF that serves as the organizer of signaling complexes in cell membranes. Sj-TSP-#3 shared a high specific domain with TSP. Moreover, Sj-TSP-#2, Sj-TSP-#4 and Sj-TSP-#5 shared a high specific domain with uroplakin-I [24]. Although Sj-TSP-#6 showed a non-specific match with tetraspanin, it had structure characteristic of 'tetraspanin' proteins. Therefore it is classified as TM4SF family. In addition, a phylogenetic tree was constructed basing on the deduced amino acid sequences of Sj-TSP-# 1~6 and their closest homologues from *S. mansoni*, including several known TSP proteins (Figure 2B). Sj-TSP-#1 shared 83% identity with CD151-related protein [Genbank: XP_002582093.1], which is a member of TM4SF [25]. Sj-TSP-#2 shared 29% with Tetraspanin-1 (Tspan-1) [Genbank: XP_002577444.1], which is a tumor proliferation-related protein from the TM4SF [26]. Sj-TSP-#3 shared 98% with tetraspanin family protein 16 invertebrate [Genbank: XP_002573925.1]. Sj-TSP-#4 shared 38% with tetraspanin-CD63 receptor [Genbank: XP_002574749.1]. Sj-TSP-#5 shared 64% with tetraspanin D76 [Genbank: XP_002575497.1], which was the same sequence of tetraspanin-18 [27]. Sj-TSP-#6 shared 82% with transmembrane 4 superfamily member [Genbank: XP_002579012.1]. These data further suggested that the six novel TSP proteins be the members of the TM4SF. In addition, the phylogenetic relationship revealed that Sj-TSP-#1 and Sj-TSP-#3 were classified into the CD subfamily while Sj-TSP-#6 was classified into the CD63 subfamily, and the rest three proteins were classified into the Uroplakin subfamily (Figure 2B).

### Analysis of transcription and expression profile of the six TSP genes at different developmental stages and genders of the parasite

**Figure 3:**
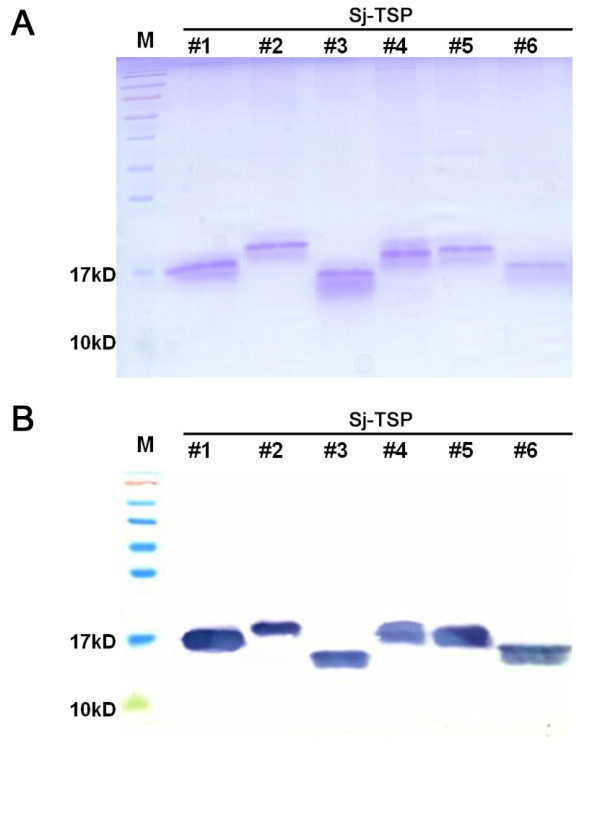
**SDS-PAGE analysis of purified recombinant proteins**. A: SDS-PAGE analysis of purified rSj-TSP-#1~6 in lane 1~6, respectively. B: western blotting of the recombinant TSP using 6-His antibody.

**Figure 4:**
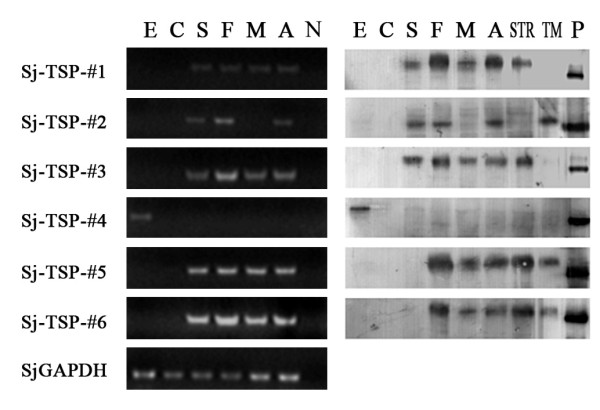
**Stage-specific expression of the TSP transcripts and proteins**. A: The mRNA levels in eggs (E), cercaria (C), liver-stage schistosomulum (S), and adult worms (A) including female (F) and male (M) determined by semi-quantitative RT-PCR. SjGAPDH gene was used as an endogenous control. No template was a negative control (N). B: Expression of the TSP proteins in various stages was detected by Western blot using anti-rSj-TSP-#1~6 mouse sera, respectively. In addition, the protein extracts were prepared in the tegument proteins (TM) and the tegument-stripped proteins (STR). The positive control rSj-TSP-#1~6 were detected to product a specific band under the reaction of each immune sera.

To examine transcriptional patterns of the six TSP members at different developmental stages and genders, the screened TSP genes were generated by RT-PCR. As shown in Figure 3A, the control gene of SjGAPDH was produced in all the stages and genders, but the six TSP genes showed different patterns of transcription: Sj-TSP-#4 was detected only in the egg stage while the other TSP genes were detected in both schistosomulum and adult parasite (Figure 4A). Interestingly, the Sj-TSP-#2 gene was transcribed in female but not in male parasites (Figure 4A).

**Figure 5:**
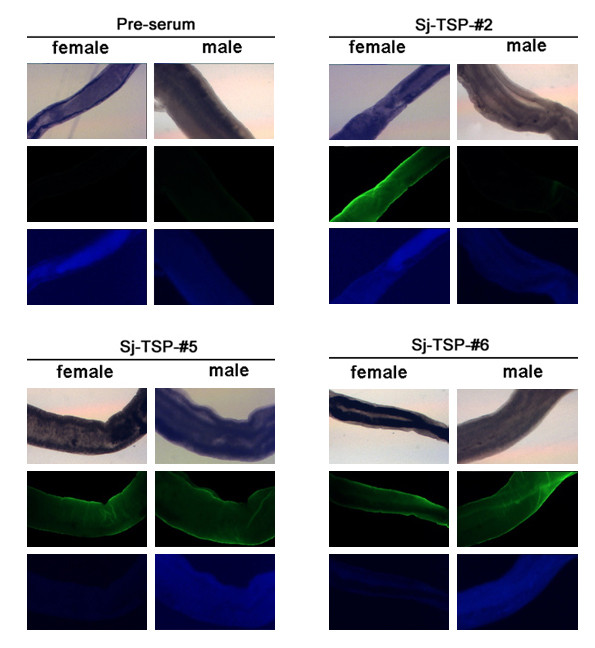
**Immunolocalization of the TSP proteins**. Immunofluorescence of adult female worms (left) and male worms (right) were performed by anti-Sj-TSP-#2, anti-Sj-TSP-#5 and anti-Sj-TSP-#6 immune sera as well as pre-immune sera followed by FITC-conjugated antibody to mouse IgG. Optical images (upper panels), corresponding fluorescence microscopy images (green, middle panels) and tissue nuclei (DAPI labeled, blue, bottom panels) of adult worms are shown. Pre-immune serum was used as negative control. A magnifications bar in panels is 100 μm.

To investigate expression profiles of the novel TSP proteins in different stages of the parasite by Western blotting, we firstly expressed the six novel TSP recombinant proteins (rSj-TSP-#1~6) in *E.coli *and purified the proteins using Ni-column. The six purified proteins were shown in Figure 3A and interacted with anti-His-tag monoclonal antibody as shown in Figure 3B. To prepare the immune sera, groups of mice were immunized with the individual rSj-TSP-#1~6, respectively. Protein extracts derived from different stages of parasite were subjected to immunoblotting and interacted with their corresponding immune sera. As shown in Figure 4B, the native Sj-TSP-#1, Sj-TSP-#2 and Sj-TSP-#3 proteins were detected in schistosomulum and adult worm stages with the exception of the male adult that does not express the Sj-TSP-#2, which is similar to the observation in transcription detection (Figure 4A, lane M). Similarly, expression of Sj-TSP-#4 was detected only in the egg stage (Figure 4B). In addition, expression of Sj-TSP-#5 and Sj-TSP-#6 were detected in the adult worm but not in schistosomulum stages (Figure 4B). Moreover, Sj-TSP-#2 expression was detected in the purified tegument component (Figure 4B, lane TM) of the adult worms while the Sj-TSP-#1 and Sj-TSP-#3 expressions were detected in tegument-stripped component (Figure 4B, lane STR) as the Non-TM TSPs, which is consistent to other observation that the Sj-TSP-#3 was expressed on the gastrodermis of *S. japonicum *[28]. And the Sj-TSP-#5 and Sj-TSP-#6 were present in both components, suggesting they were located on the surface and in internal tissues of the adult worms as well (Figure 4B). We performed immunofluorescence assays to determine localization of the proteins using various immune sera. As shown in Figure 5, Sj-TSP-#2, Sj-TSP-#5 and Sj-TSP-#6 proteins as TM TSPs were located in the tegument on whole-mount adult worms of *S.japonicum*.

## Discussion

Genetic evidence found in fungi, worms, and mammals confirmed that TSP exerts a wide influence in the coordination of intracellular and intercellular processes, including host-parasite interaction which may be associated with immune evasion [14]. Great success has been achieved in vaccinating against schistosomiasis using TSP recombinant proteins such as SmTSP2 [18]. It appears that TSPs are the most important proteins which have been found in schistosomes, and identification of novel TSP proteins in *S. japonicum *can help to develop new tools for the control of schistosomiasis. In this work, we screened and identified six novel TSP genes in *S. japonicum *and analyzed their expression profile during various developmental stages of the parasite. Availability of these novel Sj-TSP members should provide a basis for further investigation of their biological functions.

TSP forms a large group of integrated-membrane proteins. The proteins in this family are 200-300 amino acids in length and have four trans-membrane domains; there is one SEL region containing 13-30 amino acids, a short intracellular sequence, and a second LEL harbored CCG motif, which is variable in sequence length in determining its functional specificity [29]. Protein palmitoylation is the modification of juxtamembrane cysteine residues [30] that results in the formation of a thioester linkage in the protein and plays a crucial role in the TSP web [31]. Conserved N-glycosylation sites are required for signal transduction, intercellular stability and subunit folding [32]. The six novel TSP proteins identified in this study have these characteristics including the size, four trans-membrane domains, the LEL with the CCG motif, and glycosylation etc. These characteristics are also used to distinguish TSPs from other four trans-membrane proteins.

In the TM4SF, the TSP proteins can be divided into four major subfamilies: the CD family, the CD63 family, the uroplakin family and the retinal-degeneration-slow (RDS) family [33]. However, the RDS-containing TSPs have not been identified in schistosomes. This could be due to lack of bioinformatical data for the cercaria stage [34]. The classification approach determined that the CD63-like group included the CD family (including CD151, CD9, TSPAN1, *et al*), the uroplakin family and the CD63 family (such as CD63, TSPAN31, *et al*), indicating that these three families likely diverged from a CD63-like ancestor [35]. Therefore, Sj-TSP-#2, Sj-TSP-#4 and Sj-TSP-#5 containing uroplakin-I-like LEL, as well as Sj-TSP-#1 (similar with CD151-related from *S. mansoni*) and Sj-TSP-#3 (a score of 124 when compared with the CD9 antigen [Genbank: NP_031683.1]), may have similar functional properties, relative to their closely CD63 proteins. Among them, Sj-TSP-#3 could be more closely related to CD9 which plays a role in the immune modulation [36] and fertilization [37]. We suppose that it had a similar function in schistosome immune evasion and reproductive biology. Sj-TSP-#6, with a score of 148 when compared with TSPAN31 [Genbank: NP_080258.1], is also included in the CD63 family of TSP based on sequence analysis [33]. Therefore, it is suggested that the six genes share a common ancestor of CD63-like group.

The RT-PCR results showed that the six TSPs have distinct expression profiles during the life cycle of the parasite excluding the cercaria stage. Their protein expression patterns also were in accordance with their transcriptions with the exception of Sj-TSP-#5 and Sj-TSP-#6 protein. Tran's experiment [38] pointed that the highest level of SmTSP1 expression was detected in cercaria whereas SmTSP2 expression was lowest in cercaria. This suggested that TSP members likely have different functions during various development stage of schistosome. In addition, the TSP proteins of the parasite can be expressed either tegument component or the tegument-stripped component or both components. As the tegument is generally viewed as the most susceptible structure to host-mediated immune attack [39-41], we would suggest that Sj-TSP-#2, Sj-TSP-#5 and Sj-TSP-#6 as TM TSPs should be considered as priority antigens for vaccine candidates. Although both Sj-TSP-#1 and Sj-TSP-#3 were non-TM TSPs, we also could not rule out their possible effects on immune modulation [42]. Recent technical advances assisting to guide TSP research focus on the LEL region of the TSP basing on its important functions in mediating protein-protein interactions [43]. Strategies for targeting functions of TSP include recombinant soluble LEL, monoclonal antibodies (mAbs), RNA interference (RNAi) and additional approaches [44]. Further investigations are still necessary to determine potential functions of the Sj-TSPs involving immune modulation and growth regulation of the parasite by various approaches including use of monoclonal antibody to the TSP proteins, and also the RNAi technique.

## Conclusions

This work demonstrated the existence of six novel TSP proteins in *S. japonicum*. The proteins showed different transcriptional and expression profiles during various developmental stages and gender. Three of the six TSP proteins were located in tegument structure. The work presented here expands future exploration into biological roles of *S. japonicum *TSP proteins.

## Methods

### Animals and parasites

Female BALB/c mice were purchased from the Chinese Academy of Sciences in Shanghai. The animals remained under specific pathogen-free conditions and had access to food and water *ad libitum*. Mice were 6-8 weeks of age at the start of each experiment, cercaria were obtained from the National Institute of Parasitic Diseases, Chinese Centre for Disease Control and Prevention in Shanghai. Live-stage schistosomulum and adult worms were harvested from rabbits by mesenteric perfusion at 3 weeks and 6 weeks, respectively. Eggs were obtained from the livers of mice 6 weeks after infection by *S. japonicum*. All experimental procedures performed on animals within this study were conducted in accordance with, and by approval of the Internal Review Board of Tongji University School of Medicine.

### Screening for new members of the tetraspanin family by bioinformatics analysis

The new nucleotide and deduced amino acid sequences of tetraspanins were analyzed from published integrated data [22]. The transmembrane domains in the proteins were predicted by THMHMM 2.0 (http://www.cbs.dtu.dk/services/TMHMM/). The N-linked glycosylation sites were predicted by Asn-X-Ser/Thr (X stands for any amino acid except for Pro) motif. The protein domain was predicted by CD-search (http://www.ncbi.nlm.nih.gov/cdd). The protein palmitoylation sites were predicted by CSS-Palm 2.0 software.

### Multiple alignments and phylogenetic analyses

Multiple alignments of new protein sequence were performed by CLUSTALW and phylogenetic tree was constructed using the Neighbour-Joining method [45] and bootstrap analysis (1000 replication) [46] in MEGA 4 software. The tree was drawn to scale, with branch lengths in the same units as those of the evolutionary distances used to infer the phylogenetic tree. The numbers of the tree represent the confidence of the branches assigned by bootstrap.

### Reverse transcription polymerase chain reaction (RT-PCR)

The new TSP gene Sj-TSP-#1~6 mRNA expression profiles in various cell lines were analyzed by RT-PCR. Total RNA was isolated from S. *japonicum *during different developmental stages using a Trizol reagent (Invitrogen), and first strand cDNA were synthesized from 1 μg of the total RNA with an oligo (dT) 15 primer (Invitrogen). The Sj-TSP-#1~6 open reading frame were amplified with their respectively specific primers based on the listed accession numbers in Tab 1. GAPDH from *S. japonicum *(SjGAPDH) primers (forward prime: 5'- GGACCATTAAAAGGCATCTTGG-3', reverse prime: 5'-GCAACTGTAGCCGAATTCATTG-3') serving as a positive control. No template was as a negative control. The PCR reaction was performed using ExTaq enzyme (Takara) and initiated with one cycle of 5 min at 94°C, followed by 26 cycles of 30 s at 94°C, 30 s at 55°C, and 40 s at 72°C. PCR products were detected from agarose gel electrophoresis.

### Recombinant protein expression and purification

The gene fragments encoding LEL of Sj-TSP-# 1~6 were amplified by PCR with Pfu polymerase. Specific forward and reverse primers with introduction of an EcoRI or HindIII site at their extremities were used for the rSj-TSP-#1~6. Recombinant plasmids pET28a+ vector (Novagen, USA) containing the specific fragment of six TSP members were constructed. After sequencing, the correct plasmids were transformed into *E.coli *BL21 strain (DE3) (Invitrogen, USA) to express. Expression of recombinant proteins was induced with IPTG at 1mM. These recombinant proteins were purified with a hexahistidine tag and confirmed by 6-His tag antibody (TIANGEN, China).

### Preparations of mouse anti-rSj-TSP-#1~6 specific antisera

To produce the antisera to the recombinant proteins, BALB/c mice were immunized subcutaneously with individual 20 μg recombinant protein by Freund's complete adjuvant (Sigma, USA), followed by two boosting with Freund's incomplete adjuvant (Sigma) at 2 weeks intervals. Polyclonal antibody sera were collected 10 days after the third immunization.

### Proteins preparation

Total proteins from eggs, cercaria, liver-stage schistosomulum, mixed adult worms, male and female adult worms were extracted in 40 mM Tris, pH 7.4, 2% SDS supplemented with proteinase inhibitors (Sigma) on ice. Moreover, the tegument extract was obtained by a freeze/thaw/vortex procedure, as previously described [47]. Briefly, frozen adult worms were thawed on ice in the presence of 1 ml RPMI 1640 medium plus proteinase inhibitors (Sigma), with 10 vortex pulses at a maximum speed for 1 s to detach the tegument. The supernatant part collected namely tegument proteins. The left tegument-stripped worms were extracted in a similar way according to the total proteins preparation. A Bradford Assay (Bio-Rad, CA, USA) was used to measure protein levels.

### Western blotting assay

Equal quantities of proteins were separated by 12% SDS-PAGE and transferred to nitrocellulose membranes (Millipore). Membranes were incubated with primary antiserum of specific rSj-TSP-#1~6 and probed with an alkaline phosphatase (AP) -conjugated goat anti-mouse IgG (H+L) antibody (Promega) for 1 h at room temperature (RT). Subsequently, the chemiluminescence AP substrate (Promega) was used to detect the specific bands. Recombinant proteins were as a positive control to product a specific band under the reaction of their respective antisera.

### Immunolocalization

Immunofluorescence assay of adult worms from *S.japonicum *was conducted as previously described [18]. Freshly female and male worms from *S. japonicum *were fixed in 100% methanol. After blocking, the sections were incubated with mouse anti-sera Sj-TSP-#2, 5, 6 (1:25 dilution) and followed by FITC-conjugated goat antibody to mouse IgG (Sigma, 1:150 dilution). Pre-vaccination serum was used as a negative control. Worms were counterstained with DAPI (Sigma; 0.1 mg/ml in PBS), which stains nuclei. Images were acquired with a fluorescence microscope.

## Competing interests

The authors declare that they have no competing interests.

## Authors' contributions

JYY conceived the project and carried out the experimental work, interpreted and analyzed the data and wrote the manuscript. XXD conceived the project, provided a resource of databases cited and some technical support. QXX provided significant support to the preparation of the recombinant proteins. PWQ conceived the project, critically revised the manuscript and gave the final approval of the version to be published. All authors read and approved the final manuscript.
